# Rational engineering of adeno‐associated virus capsid enhances human hepatocyte tropism and reduces immunogenicity

**DOI:** 10.1111/cpr.13339

**Published:** 2022-09-22

**Authors:** Jiabao Han, Liyu Zhu, Jingwen Zhang, Lu Guo, Xuehan Sun, Cheng Huang, Kai Xu, Ying Zhang, Wei Li, Qi Zhou

**Affiliations:** ^1^ State Key Laboratory of Stem Cell and Reproductive Biology Institute of Zoology, Chinese Academy of Sciences Beijing China; ^2^ Institute for Stem Cell and Regenerative Medicine, Chinese Academy of Sciences Beijing China; ^3^ University of Chinese Academy of Sciences Beijing China; ^4^ College of Life Science, Nankai University Tianjin China; ^5^ Beijing Institute for Stem Cell and Regenerative Medicine Beijing China

## Abstract

**Objectives:**

Gene therapy based on recombinant adeno‐associated viral (rAAV) vectors has been proved to be clinically effective for genetic diseases. However, there are still some limitations, including possible safety concerns for high dose delivery and a decreasing number of target patients caused by the high prevalence of pre‐existing neutralizing antibodies, hindering its application. Herein, we explored whether there was an engineering strategy that can obtain mutants with enhanced transduction efficiency coupled with reduced immunogenicity.

**Methods:**

We described a new strategy for AAV capsids engineering by combining alterations of N‐linked glycosylation and the mutation of PLA2‐like motif. With this combined strategy, we generated novel variants derived from AAV8 and AAVS3.

**Results:**

The variants mediated higher transduction efficiency in human liver carcinoma cell lines and human primary hepatocytes as well as other human tissue cell lines. Importantly, all the variants screened out showed lower sensitivity to neutralizing antibody in vitro and in vivo. Moreover, the in vivo antibody profiles of variants were different from their parental AAV capsids.

**Conclusions:**

Our work proposed a new combined engineering strategy and engineered two liver‐tropic AAVs. We also obtained several AAV variants with a higher transduction efficiency and lower sensitivity of neutralizing antibodies. By expanding the gene delivery toolbox, these variants may further facilitate the success of AAV gene therapy.

## Graphical abstract

Transduction efficiency and immune responses hinder the application of recombinant adeno‐associated viral (rAAV) vectors in gene therapy. Here, Han et al. describe a new combined strategy for AAV capsid engineering and obtain several AAV variants with higher liver transduction efficiency and less immune activation.

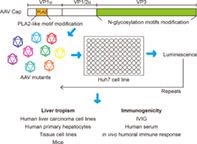

## 1 INTRODUCTION

Gene therapy offers potential strategies for the treatment of genetic diseases that are difficult to target with traditional therapies. At present, gene therapy is mainly achieved through the persistent expression of therapeutic genes or “transfer genes”, so that their expression level can improve or cure disease symptoms.^1^ This can be achieved in two different ways: one strategy is to introduce the integration vector into precursor cells or stem cells, whereby the gene will be passaged to each daughter cell^2^, ^3^, ^4^; the other strategy is to transfer the target gene into nondividing cells by a nonintegrated vector, allowing the long‐term expression of the target gene in the whole cellular life cycle.^5^, ^6^, ^7^, ^8^, ^9^ Vectors for in vivo gene delivery usually include viral vectors, such as adenoviral vectors, and recombinant adeno‐associated viral (rAAV) vectors, and nonviral vectors, such as lipid nanoparticles (LNPs) and exosomes.^10^, ^11^, ^12^ The recombinant AAV vector is modified from a nonpathogenic and nonenveloped parvovirus.^13^ Most of the rAAV vector's DNA persists in the cell as episomes and will not be integrated into patients' genome. Therefore, the risk of insertion mutations into human genome is low.^14^ Considering these strengths, rAAV vector is a leading gene delivery system in gene therapy being widely used in preclinical and clinical researches. Currently, dozens of gene therapy based on rAAV vectors are under clinical trials, aiming to treat complicated diseases, including neuromuscular, central nervous system, ophthalmological, and liver diseases (reviewed in Ref. 15).

Although 13 human and nonhuman primate AAV serotypes and over 150 AAV variants have been identified with different tropisms,^16^, ^17^ AAV, serving as a carrier of gene therapy, still has some shortcomings. One of the key limitations in clinical applications is the adaptive immune response,^18^ which includes two different types: humoral immune and cellular immune response.^19^, ^20^, ^21^ Humoral immune responses hamper the accessibility of rAAV to target tissues caused by a high prevalence of pre‐existing neutralizing antibodies in the population.^22^, ^23^, ^24^ Cellular immune responses to rAAV are mainly considered of deleterious capsid cytotoxic T lymphocyte (CTL), causing loss of transgene expressions and potentially liver toxicities. Early clinical trials have found these adverse events are correlated with CD8+ T cell responses against the rAAV capsid.^25^, ^26^ These adverse effects appear to a dose‐dependent trend.^27^ Later studies have also shown that cytotoxic CD8+ T cells recognize capsid‐derived epitopes via major histocompatibility complex class I (MHC class I) on the transduced cells, and partly due to high abundance of unmethylated CpG motifs in the viral genome.^28^, ^29^ Therefore, avoiding pre‐existing neutralizing antibodies and controlling the dose of injection are keys to develop AAV for clinical treatments.

Reducing the sensitivity of pre‐existing neutralizing antibodies and improving the transduction efficiency of AAV to histocytes to reduce the injection dose and the immunogenicity of AAV could be achieved by an engineering strategy. Glycosylation plays an important role in the viral life cycle.^30^, ^31^, ^32^, ^33^ Several studies have shown that N‐linked glycosylation of viral surface proteins affects the infection process mainly by affecting the binding of the virus to relevant receptors on the host cell surface.^34^, ^35^, ^36^ A previous study used AAV2, with the modified glycosylation of its capsid proteins and obtained mutant viruses with higher gene expression levels than those of the unmodified viruses.^37^

The capsids of AAV contain VP1, VP2 and VP3, these VPs overlap in sequence while VP1 containing a VP1‐unique (VP1u) region. Several functional elements, such as the conserved phospholipase A2 (PLA2)‐like motif and the nuclear localization signal (NLS) in parvoviruses, have been identified in VP1/2.^38^ The PLA2‐like motif was considered to be critical for viral escape from endosomes to the nucleus through conformational changes of AAV capsids.^39^, ^40^, ^41^ As the PLA2‐like motif activity of AAV is lower than other parvoviruses, it is unknown whether increasing the PLA2‐like activity can improve the efficiency for transgene expression of rAAV.^42^

Here, we propose a new combined strategy to modify both the N‐linked glycosylation sites of the AAV capsid proteins and the PLA2‐like motif. AAV8 is one of the most widely used AAV variants in liver targeting, and a recently engineered variant, AAVS3, shows strong transduction efficiency in clinical trials.^43^, ^44^, ^45^ AAVS3 displays higher transduction efficiency in primary human hepatocytes, while, as an AAV3B‐derived vector, it may also show a higher prevalence of pre‐existing neutralizing antibodies in the population.^46^ Hence, we chose AAV8 and AAVS3 to determine whether our strategy can obtain novel variants with enhanced transduction efficiency coupled with lower sensitivity to pre‐existing neutralizing antibodies.

## 2 MATERIALS AND METHODS

### 2.1 Generation of AAVs for mutation site selection

AAVs were packaged using a two‐plasmid system, consisting of an AAV vector packaging plasmid (pDP2/8 or pDP2/S3) and an expression plasmid (px602‐CMV‐Luciferase‐green fluorescent protein [GFP]). 2 μg expression plasmids and 8 μg packaging plasmids were transfected into 10^7^ HEK293T (Procell, CL‐0005) seeded 24 h before transfection using Lipofectamine LTX Reagent with PLUS Reagent (Thermo Fisher Scientific, 15,338,100) according to the manufacturer's instructions. The supernatant was collected 48 and 72 h after transfection and cells were digested, lysed, and mixed with supernatant. More detailed methods for lab‐grade AAV production and AAV titration were described in Supplementary Methods.

### 2.2 Sequences and mutated sites

The mutation sites of each of the mutants are described in Supplementary Methods.

### 2.3 sPLA2 catalytic activity assay

1 × 10^12^ vg AAV virions were incubated at 65°C for 10 min. The activity of PLA2‐like motif was assayed using the Secretory Phospholipase A2 Assay Kit (Abcam, ab133089) according to the manufacturer's instructions.

### 2.4 Animals

The 8‐week‐old C57BL/6J male mice used for evaluation of the in vivo experiments were purchased from Beijing Vital River Laboratory Animal Technology Co., Ltd.

### 2.5 Cell culture conditions

HEK293T, Huh7 (Procell, CL‐0120), HepG2 (ATCC, HB‐8065), Hep3B (Procell, CL‐0102), MRC‐5 (Procell, CL‐0161), HL‐1 (Procell, CL‐0605), AC16 (BeNa Culture Collection, BNCC337712), Hepa 1–6 (Procell, CL‐0105), and Renca (Procell, CL‐0568) cells were cultured in Dulbecco's Modified Eagle Medium (DMEM) (Gibco, 11,965,084) with 10% fetal bovine serum (FBS) (Gibco, 10,099,141), 1% GlutaMAX supplement (Gibco, 35,050,061), and 1% Antibiotic‐Antimycotic (Gibco, 15,240,062). Human primary hepatocytes were purchased from bioIVT and thawed and plated according to the manufacturer's instructions.

### 2.6 Detection of transduction efficiency

Cells were seeded into a white/clear 96‐well plate (Corning Mediatech, 3903) with 2 × 10^4^ cells per well 24 h before transduction. AAVs were transduced at different multiplicity of infection (MOIs). The luciferase assay kit was used to detect luciferase activity according to the manufacturer's instructions (Bright‐Lite Luciferase Assay System, Vazyme, DD1204).

### 2.7 Evaluation of transduction efficiency in multiple tissue cell lines

Human cell lines HL‐1, HEK293T, and MRC‐5 were seeded on tissue‐culture (TC)‐treated circular glass coverslips (Solarbio, YA0353) in 4‐well plates at 4 × 10^4^ cells per well 24 h before transduction, and transduced with variants and parental AAVs at MOIs of 1 × 10^4^ and 1 × 10^5^ for each cell line, respectively. Cells were fixed 72 h after transduction in a 1% paraformaldehyde fix solution (Solarbio, P1111) at room temperature for 20 min, and then washed with phosphate buffered saline (PBS). Fixed cells were permeabilized with PBS containing 0.2% Triton X‐100 at room temperature for 30 min, washed with PBST (PBS + 0.1% Tween 20), and blocked with 1% bovine serum albumin (BSA) and 22.52 mg/ml glycine in PBST at room temperature for 1 h. An anti‐GFP antibody (Abcam, ab13970) was diluted with 1% BSA at 1:2000 and used to incubate the samples at room temperature for 2 h. After washing with PBS, a secondary antibody for goat anti‐chicken Alexa Fluor 488 IgG antibody (Thermo Fisher Scientific, A11039) was diluted with 1% BSA at 1:500, and incubated at room temperature for 1 h in the dark. Cells were washed with PBS, and incubated with a 4′,6‐diamidino‐2‐phenylindole (DAPI) staining solution (Abcam, b228549) in a ratio of 1:1000 for 3 min. All images were collected using a Zeiss LSM 880 confocal microscope. The number of GFP positive cells was counted by ImageJ.^47^ The transduction methods for human primary hepatocytes were described in Supplementary Methods.

### 2.8 In vitro neutralizing antibody titration

Huh7 cells were seeded in white/clear 96‐well tissue culture plates at 1 × 10^4^ per well and incubated overnight. Each variant and the parental AAV containing a CMV‐Luciferase‐GFP cassette was diluted to 4.8 × 10^10^ vg/ml with PBS. Human Immunoglobulin for Intravenous Injection (IVIG, HUALAN BIOLOGICAL ENGINEERING CHONGQING CO., LTD, S20113011) was diluted with heat‐inactivated FBS in an adequate range and incubated with each AAV vector at 37°C for 1 h. Huh7 cells were transduced with the mixture in triplicate at an MOI of 1 × 10^4^. Luciferase activity was determined 72 h after transduction using the luciferase assay kit according to the manufacturer's instructions (Bright‐Lite Luciferase Assay System, Vazyme, DD1204). The human serum (Normal human serum [mixed], Lablead, 9193) neutralizing antibody evasion capacity detection method was the same as that for IVIGs detection. For mice serum, blood was collected from the submandibular vein and incubated at room temperature for 30 min. The clot was removed by centrifuging at 3500 rpm for 15 min at room temperature. After centrifugation, the supernatant was collected and detected.

### 2.9 In vivo analyzing biodistributions and antibody profiles

In vivo biodistribution of the AAV8 mutants in C57BL/6J mice. We tested the biodistribution of five AAV8 candidate mutants in 8‐week‐old wild‐type C57BL/6J male mice. The variants contained a CMV‐Luciferase‐GFP cassette, and the parental AAV8 contained CMV‐mCherry‐Luciferase cassette. We mixed each mutant virus with WT virus in a 1:1 ratio to produce a total viral dose of 1 × 10^12^ vg and diluted the volume to 200 μl. The mixed virus was intravenously injected into 8‐week‐old male C57BL/6J mice via the tail vein. Mice were sacrificed and perfused by PBS 3 weeks after injection. The DNA and RNA of the heart, liver, spleen, lung, and kidney were isolated to determine the copy number of GFP and mCherry to calculate mutant virus frequency in each tissue. cDNA was produced by reverse transcription (RT) using the reverse transcription assay kit according to the manufacturer's instructions (HiScript III RT SuperMix for qPCR, Vazyme, R323). Primer and probe sequences for GFP were GFP‐F: TCCGCCACAACATCGAGGAC, GFP‐R: GTAGTGGTTGTCGGGCAGCA, and GFP‐P: 6‐FAM‐CAGCGTGCAGCTCGCCGACC‐BHQ. Primer and probe sequences for mCherry were mCherry‐F: CAGAGGCTGAAGCTGAAGGA, mCherry‐R: GCTTCTTGGCCTTGTAGGTG, and mCherry‐P: 6‐FAM‐CGGCGGCCACTACGACGCTG‐BHQ. We considered the ratio of GFP/mCherry in the tissue as the tissue frequency, the ratio of GFP/mCherry in the mixed virus as the virus frequency, and the ratio of tissue frequency/virus frequency as the mutant virus frequency in this tissue.

In vivo antibody profiles of the AAV8 mutants in C57BL/6J mice. Eight‐week‐old C57BL/6J male mice were injected intravenously with 5 × 10^11^ vg AAV8 virions. Two weeks after treatment, five variants and AAV8 vector contained luciferase cassette were dosed with 5 × 10^11^ vg to pre‐treated mice as three mice per variants. One week after re‐administration, animals were injected of luciferin substrate as 150 mg/kg per mouse. Ten minutes after luciferin injection, the mice were anesthetized with 2% isoflurane and analyzed luciferase expression by Xenogen IVIS200 imaging system and the software Living Image 4.0 (Caliper).

### 2.10 Statistics

GraphPad Prism software (GraphPad, La Jolla, CA) was used to analyze experimental values via one‐way ANOVA using Dunnett's multiple comparison test, two‐way ANOVA using Dunnett's multiple comparison test, and two‐way ANOVA using Sidak's multiple comparison test. *p* values <0.05 were considered statistically significant. *p < 0.05, **p < 0.01, ***p < 0.001, ****p < 0.0001.

## 3 RESULTS

### 3.1 Construction of AAV8 and AAVS3 variants

To verify that modifications in N‐linked glycosylation and the PLA2‐like motif could simultaneously improve AAV transduction efficiency and reduce sensitivity to pre‐existing neutralizing antibodies, two liver‐tropic serotypes, AAV8 and AAVS3, were engineered. The PLA2‐like motif on VP1u mainly affects the efficiency of endosome escape after AAV's cellular entry. N‐linked glycosylation on the surface of the viral capsid not only improve the binding efficiency of AAV to the cells but also aid its bypassing of neutralizing antibodies. Based on the above hypothesis, we performed single‐amino‐acid mutations in VP1u and VP3 (Tables S1 and S2).

We compared the sequences of VP1/2u in several common used AAV serotypes and artificially modified variants (Figure S1) as well as sequence alignment with other viruses of the family *Parvoviridae*, which perform higher PLA2 activity.^42^ Previous studies have reported some core structures and sites that are critical for the activity of the domain, which include the “HD” PLA2 motif, G54, D69, D97, H38, and H95. These motifs and sites are associated with enzyme activity and the mutagenesis on these sites can easily cause the loss of function of the enzyme. Thus, we avoided these sites as much as possible.^48^ Among several commonly used AAV vectors, AAV5, which is evolutionarily distant from others, has unique serotype properties.^49^, ^50^ Previous studies claim that substituting VP1u of AAV2 with VP1u of AAV5 can efficiently infect cells in the absence of GPR108, which is required for other AAVs during endosomal escape.^51^ Therefore, we selected amino acid sites near known active sites that were different from AAV5. Considering the above reasons, we decided to modify F56 and A98 of the PLA2 domain. The mutated sites on VP3 were selected according to the N‐linked glycosylation modification target sequence NXS/NXT.^52^, ^53^ The mutation strategy of VP3 was classified into two categories: the first was increasing N‐linked glycosylation by single‐amino‐acid mutations from non‐NXS/NXT sequences to NXS/NXT sequences and vice versa, and the second was to remove N‐linked glycosylation. Amino acid changes were based on property similarities: the mutated amino acid should be as similar as possible in polarity, charge, R‐group size, etc.

### 3.2 Selection of AAV8 and AAVS3 variants in the Huh7 cell line

**FIGURE 1:**
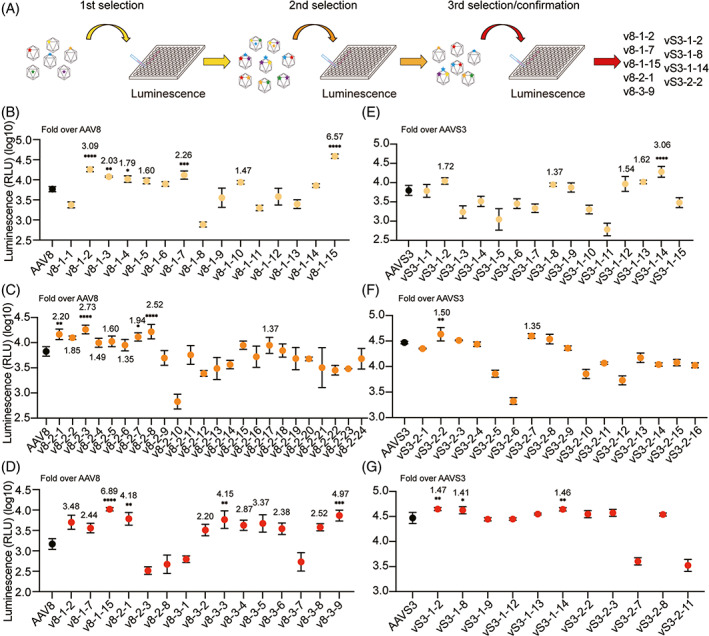
Selection of AAV8 and AAVS3 variants in the Huh7 cell line with a CMV‐Luciferase‐GFP cassette at an MOI of 100,000. (A) Workflow of multi‐round selections. (B) Huh7 cells were transduced with single‐amino‐acid mutants of AAV8. (C) The second‐round selection of AAV8 mutants. (D) The third‐round selection of AAV8 mutants. (E) Huh7 cells were transduced with single‐amino‐acid mutants of AAVS3. (F) The second‐round selection of AAVS3 mutants. (G) Repeat experiments for AAVS3 candidates. The Y‐axis showed the intensity of the luminescence by relative luminescence units (RLU). Points represented the mean of 3 replicates and error bars represented the SD (standard deviation). Experimental values were analyzed via one‐way ANOVA using Dunnett's multiple comparison test and only statistically significant differences were indicated. *p < 0.05, **p < 0.01, ***p < 0.001, ****p < 0.0001

To validate our engineering hypothesis, we carried out three rounds of selection (Figure 1A). We first engineered AAV8 and constructed 15 single‐amino‐acid mutated packaging plasmids (Table S1). All these packaging plasmids and the packaging plasmid of AAV8 were used to produce AAV vectors containing a CMV‐Luciferase‐GFP expression cassette. Huh7 cells were, respectively, transduced at a low (1 × 10^4^) and high (1 × 10^5^) multiplicity of infection (MOI), and the luciferase activity was detected at 72 h after incubation for the evaluation of transduction efficiency (Figures 1B and S2A).

Based on the different levels of luciferase activities for single‐amino‐acid mutations, we selected three promising sites (T332N, Q589N, and N665Q) to iteratively generate double‐amino‐acid and triple‐amino‐acid mutated variants. We combined these three sites into double or triple mutations and combined them with the positions selected in VP1u (F56 and A98). Using the same method and strategy, 24 double‐site mutants and nine triple‐site mutants were constructed for the second and third rounds of screening (Figures 1C, D and S2B, C). We constructed a total of 48 mutants, including single‐site and multiple‐site mutated variants, and evaluated their transduction efficiency by luciferase activity detecting assay. According to luminescence intensity, we finally selected five mutants as candidates for further validation, named v8‐1‐2, v8‐1‐7, v8‐1‐15, v8‐2‐1, and v8‐3‐9.

Meanwhile, a similar engineering strategy was used to generate AAVS3 mutants. A total of 31 AAVS3 mutants with single‐site, double‐site, and triple‐site mutated sites were constructed (Figures 1E–G, S2D–F and Table S2). We finally selected vS3‐1‐2, vS3‐1‐8, vS3‐1‐14, and vS3‐2‐2 for further verification. The mutated residues T451N, Q585N, N497Q, A98R, and N457Q were contained in these variants.

### 3.3 Characterization of sPLA2 motif catalytic activity and N‐linked glycosylation

To test whether our modification of AAV indeed increased the activity of PLA2‐like motif, we tested the PLA2 activity in several mutated variants. We found slightly elevated enzyme activity in which PLA2‐like motif was modified mutants v8‐2‐1 (A98R), v8‐3‐9 (A98E + N665Q) and vS3‐2‐2 (A98R + N457Q), while activity was undetectable in most samples (Tables S3 and S4). This may be due to the low PLA2 activity of AAV.^42^

Furthermore, characterization of N‐linked glycans on the AAV capsid was identified. For AAV8‐derived mutants, v8‐1‐2 (T332N), v8‐1‐7 (Q589N), v8‐1‐15 (N665Q) and v8‐3‐9 (A98E + N665Q), were involved in changes of N‐linked glycosylation, among which v8‐1‐2 (T332N) and v8‐1‐7 (Q589N) theoretically increases glycosylation, while v8‐1‐15 (N665Q) and v8‐3‐9 (A98E + N665Q) were designed to delete modification. Through LC/MC experiments, we found that the status of modification on AAV8 capsid at the three sites mentioned above was consistent with the hypothesis, as residues T332 and Q589 were not glycosylated while residue N665 was (Table 1). As we expected, v8‐1‐15 (N665Q) and v8‐3‐9 (A98E + N665Q), which were designed to remove glycosylation at residue N665, had no detectable glycosylation on this site. However, for v8‐1‐2 (T332N) and v8‐1‐7 (Q589N), the two designed to add glycosylation at residues T332 or Q589, respectively, did not show the corresponding modification at mutated sites. Similar to AAV8, the modification of AAVS3 at selected sites was also consistent with our hypothesis, as residues T451 and Q585 were not glycosylated while residues N457 and N497 were (Table 1). For vS3‐1‐14 (N457Q) and vS3‐2‐2 (N497Q), the glycosylation was eliminated at these sites, respectively. And residue 451 was glycosylated in variant vS3‐1‐2 (T451N) whereas vS3‐1‐8 (Q585N) did not add glycosylation at residue 585. Because not all the N‐X‐T, N‐X‐S or N‐X‐ motifs would be glycosylated in vivo, it was easy to understand that comparing with adding extra potential N‐glycosylation sites, the elimination of glycosylated sites were more likely to meet expectations.^54^, ^55^, ^56^, ^57^

**TABLE 1:** N‐linked glycans identified on modified sites by LC–MS/MS analysis

Variant	Peptide	Position of modification	Modification type (s)	Observed m/z	Observed (M + H)	Calc. Mass (M + H)
AAV8	K.NTPVPADPPTTFN[+2.988]QSK.L	665	N[+3]	858.922	1716.837	1716.832
AAVS3	Y.LNRTQGTTSGTTN[+2.988]QSRLLF.S	457	N[+3]	1049.545	2098.083	2098.077
	K.TANDNN[+2.988]NSNFPWTAASK.Y	497	N[+3]	618.943	1854.815	1854.814
vS3‐1‐2	Y.YLNRTQGN[+2.988]TSGTTNQSRL.L	451	N[+3]	1007.507	2014.006	2013.983

### 3.4 Production efficiency of the mutated capsids

**FIGURE 2:**
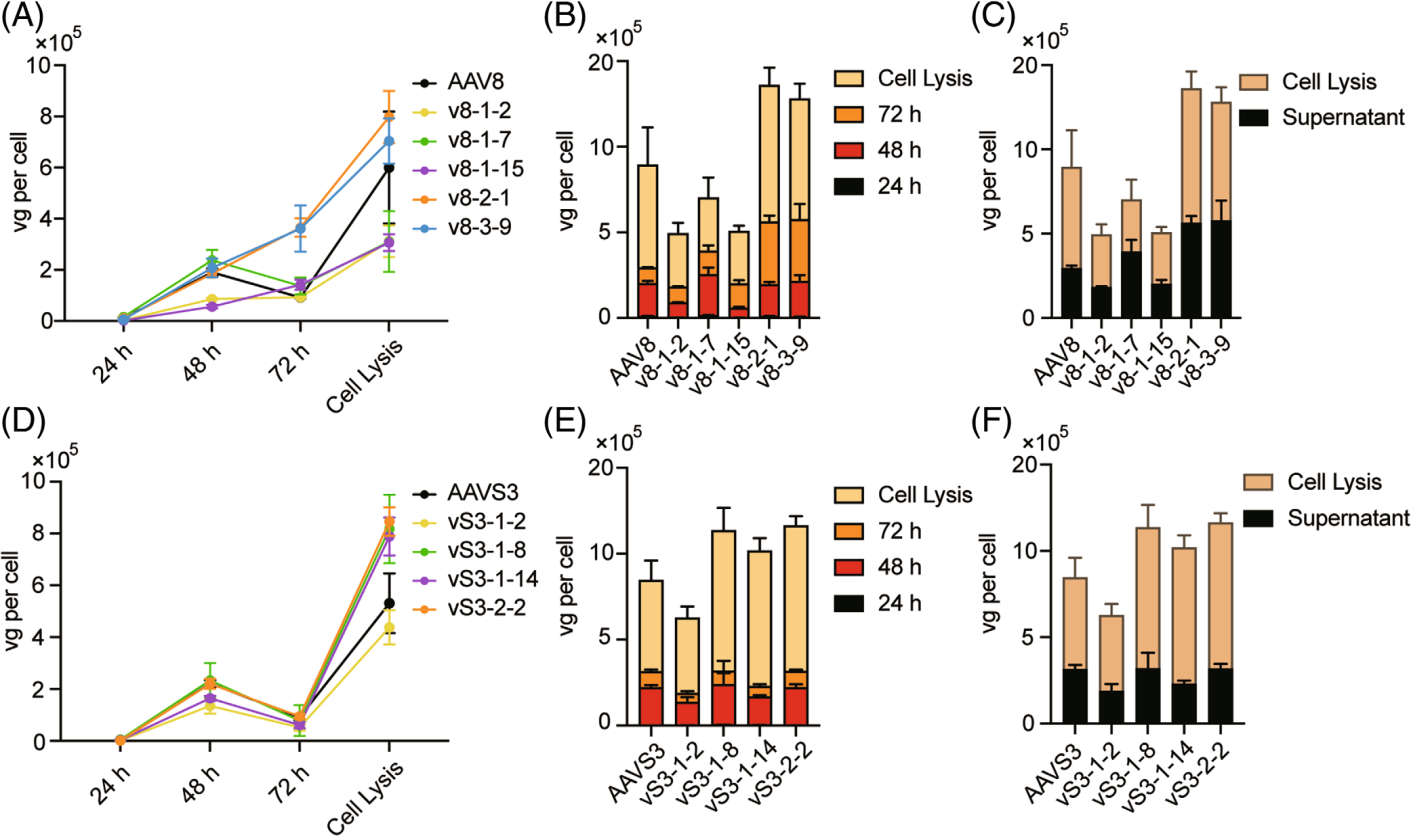
Evaluation of AAV variant packaging efficiency. (A) Viral copies of AAV8 variants and the parental AAV8 in supernatants and cell lysates. (B) Total amount and proportions of the viral copies in each period for AAV8 variants and the parental AAV8. (C) Ratio of viral amount in supernatant and cell lysates for AAV8 variants and AAV8. (D) Viral copies of AAVS3 variants and parental AAVS3 in supernatants and cell lysates. (E) Total amount and proportions of the viral copies in each period for AAVS3 variants and AAVS3. (F) Ratio of viral amount in supernatant and cell lysates for AAVS3 variants and AAVS3. Each data point represents the mean value of 3 replicates and error bars represent the SD

To test whether the mutagenesis would affect the production efficiency, we produced all the 5 AAV8 variants and 4 AAVS3 variants as well as their parental AAVs (Figure 2). HEK293T cells were used for AAV vector production and were transfected with two‐plasmid packaging system in triplicate wells for each variant. Supernatants were collected at 24 and 48 h after transfection and added fresh medium. The supernatant was also collected at 72 h after transfection and the cells were digested and lysed to determine the intracellular viral particles. The viral titration was tested by qPCR for each sample as described in Supplementary Methods. The results showed that these variants did not decrease production efficiency compared with their parental capsids, and the ratio of viral particles in the supernatant and cell lysate was also similar to the parental AAV (Figure 2C, F). Analysis of AAV8, v8‐1‐15, AAVS3, and vS3‐1‐14 by transmission electron microscopy (TEM) showed no significant difference of full/empty ratio between the mutants and parental AAV vectors (AAV8 ~ 10%, v8‐1‐15 ~ 15%, AAVS3 ~ 20% and vS3‐1‐14 ~ 17%) (Figure S3)

### 3.5 Validation of AAV8 and AAVS3 variants in multiple human liver carcinoma cell lines and human primary hepatocytes

**FIGURE 3:**
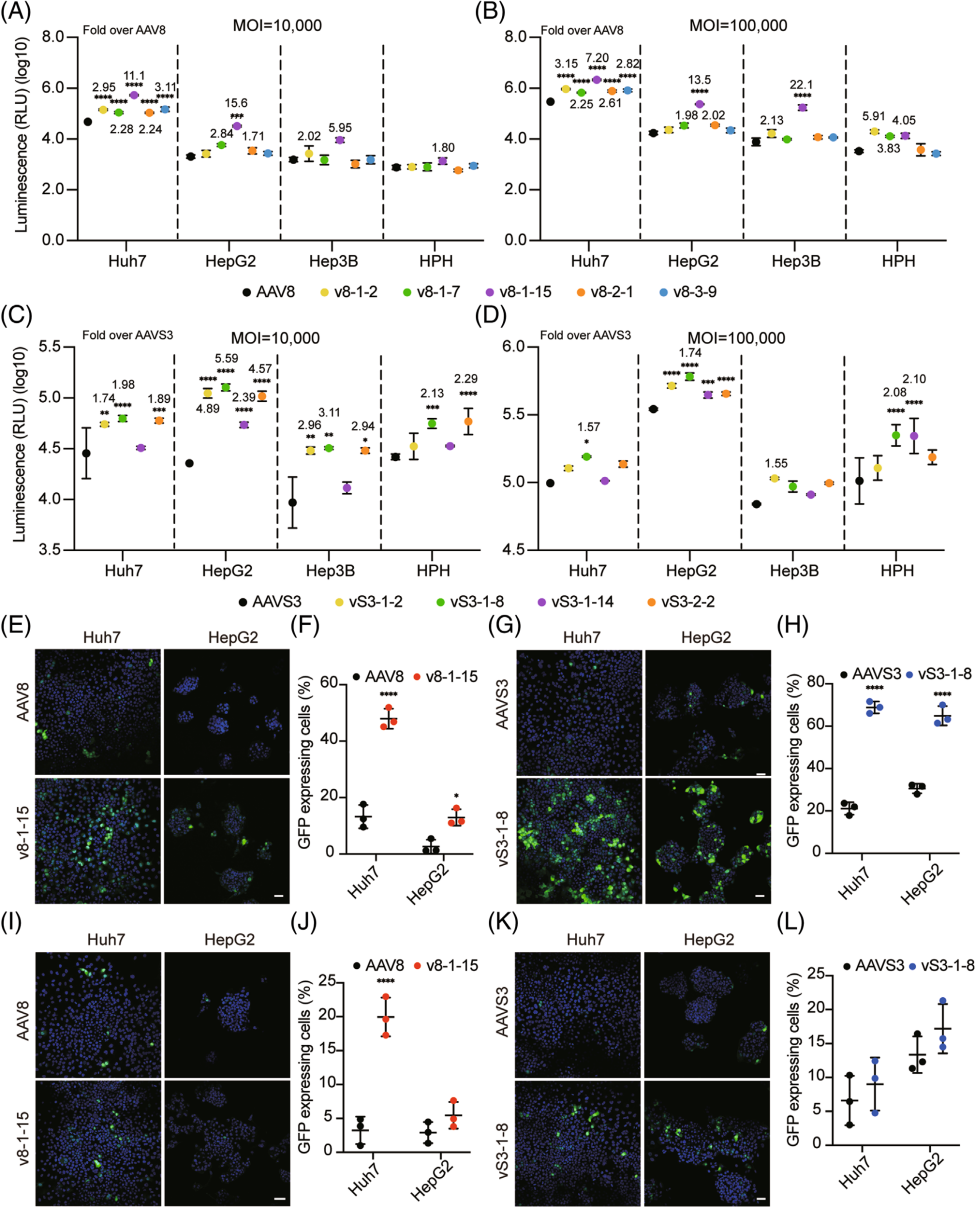
Evaluation of AAV mutants for multiple human liver carcinoma cell lines and human primary hepatocytes. (A, B) Huh7, HepG2, Hep3B, and human primary hepatocytes were transduced with the different AAV8 variants. (C, D) Cells were transduced with the different AAVS3 variants. The y‐axis showed the intensity of luminescence by relative luminescence units. Points represented the mean of 3 replicates and error bars represented the SD. Experimental values were analyzed via two‐way ANOVA using Dunnett's multiple comparison test and only statistically significant differences were indicated. (E, F) The variant v8‐1‐15 contained a CMV‐GFP cassette as well as the parental AAV8 at an MOI of 100,000 and the quantification of transduction efficiency was calculated. (G, H) Immunofluorescence for the variant vS3‐1‐8 and AAVS3 at an MOI of 100,000. (I–L) Cells were transduced at an MOI of 10,000 with different variants. Nuclei were stained with DAPI (blue) and transduction efficiency was assessed by GFP (green). Scale bar: 50 μM. The numbers of whole cells and GFP‐positive cells were quantified by ImageJ. Each data point represented an area for each sample. At least three areas were analyzed for each sample. Points represent the mean of replicates and error bars represent the SD. Experimental values were analyzed via two‐way ANOVA using Sidak's multiple comparison test and only statistically significant differences were indicated. *p < 0.05, **p < 0.01, ***p < 0.001, ****p < 0.0001

To further verify the improved transduction efficiency of our mutants, different liver cells lines, HepG2, Hep3B, Huh7, and human primary hepatocytes were transduced with laboratory‐grade purified AAVs carrying a CMV‐Luciferase‐GFP cassette. All the 5 AAV8 mutants displayed a higher transduction efficiency especially the variant v8‐1‐15 (Figures 3A, B and S4). Similarly, all 4 AAVS3 mutants showed better transduction capacity compared with the parental AAVS3 (Figure 3C, D). To confirm our results, the variants v8‐1‐15 and vS3‐1‐8 were selected for immunofluorescence. Two human liver carcinoma cell lines, Huh7 and HepG2, were transduced with different GFP‐expressing AAV vectors. Consistent with the luciferase assay results, the two variants obtained more GFP‐positive cells than their parental AAVs, indicating their higher transduction efficiency (Figure 3E–L).

### 3.6 Transduction efficiency assessment of AAV8 and AAVS3 variants in multiple human‐ and mouse‐derived tissue cell lines

Apart from the liver, other organs like the heart, kidney, brain, and lung also serve as gene therapy targets. Therefore, we also evaluated the delivery efficiency of our variants in these tissues. Cell lines of liver, heart, kidney, and lung were transduced with GFP‐contained AAV, and further underwent immunofluorescence staining to detect the transduction efficiency.

**FIGURE 4:**
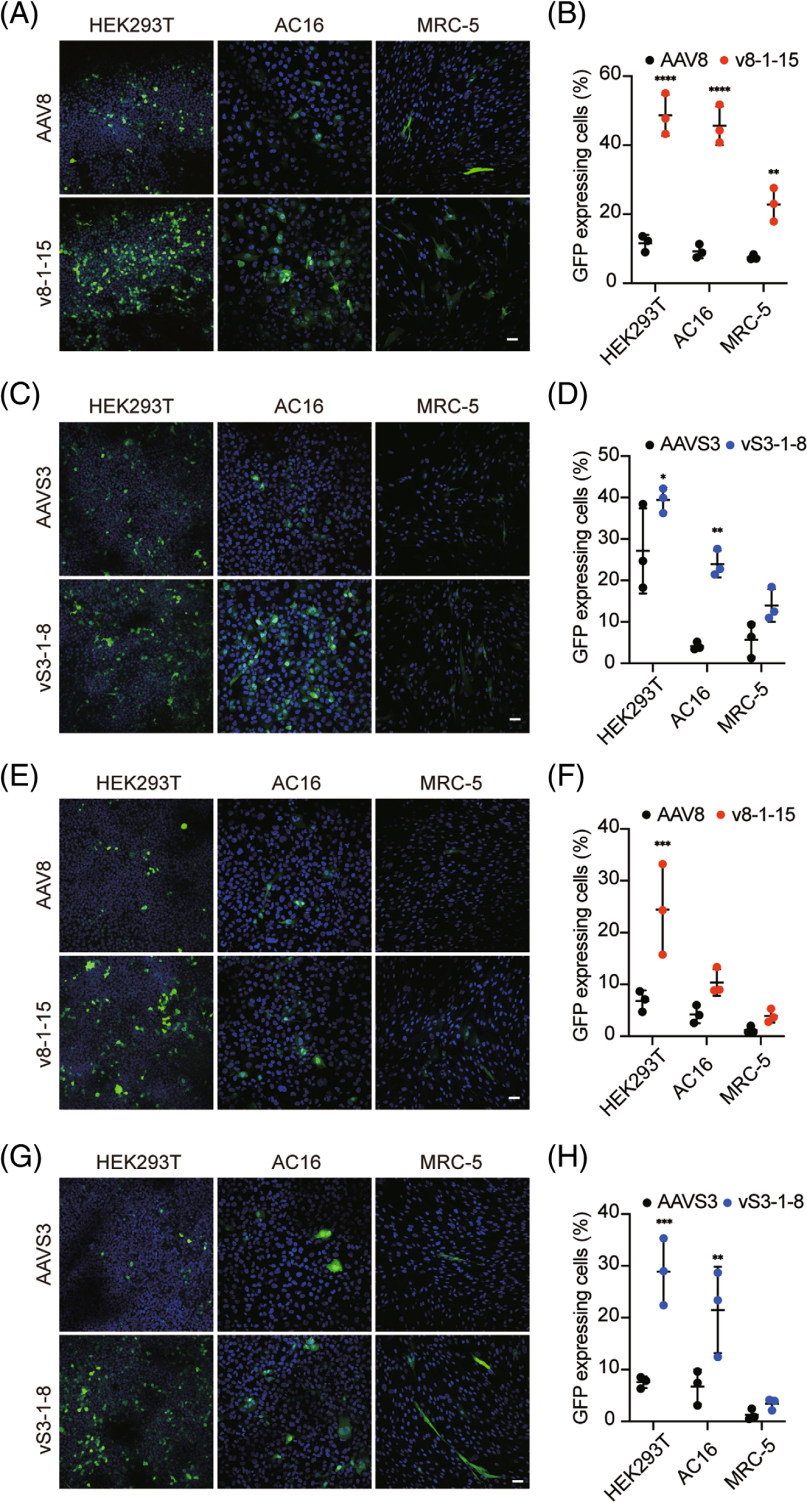
Evaluation of transduction efficiency on multiple human‐derived tissue cell lines. (A–D) Transduction of AAV8, v8‐1‐15, AAVS3 and vS3‐1‐8 at an MOI at 100,000 and quantified the efficiency. (E–H) Transduction of AAV8, v8‐1‐15, AAVS3 and vS3‐1‐8 at an MOI at 10,000 and quantified the efficiency. Nuclei were stained with DAPI (blue) and transduction efficiency was assessed by GFP (green). Scale bar: 50 μM. Each data point represented an area for each sample. At least three areas were analyzed for each sample. Points represent the mean of replicates and error bars represent the SD. Experimental values were analyze via 2‐way ANOVA using Sidak's multiple comparison test and only statistically significant differences were indicated. *p < 0.05, **p < 0.01, ***p < 0.001, ****p < 0.0001

In HEK293T (human embryonic kidney cell line), AC16 (human cardiomyocyte cell line), and MRC‐5 (human fetal lung fibroblast cell line), v8‐1‐15 and vS3‐1‐8 showed a higher transduction efficiency (Figure 4). However, in mouse‐derived cell lines Hepa 1–6 (murine liver cancer cell line), HL‐1 (immortalized murine cardiomyocyte cell line), and Renca (murine renal carcinoma cell line), the variant v8‐1‐15 did not show a higher transduction efficiency (Figure S5).

### 3.7 In vivo biodistribution of the AAV8 mutants in C57BL/6J mice

As the tissue distribution of AAV vectors affects safety and efficiency in preclinical and clinical studies, the biodistribution of new variants needed to be further evaluated. To clarify the in vivo tissue distribution, we determined the transduction profiles of our 5 AAV8 mutants in C57BL/6J mice.

All 5 AAV8 variants were packaged in a CMV‐GFP cassette, while the parental capsid AAV8 was packaged in a CMV‐mCherry cassette. Each variant was mixed with parental capsid AAV8 in a ratio of 1:1 for a total of 1 × 10^12^ vg. Three eight‐week‐old C57BL/6J male mice were injected i.v. with each mixture vectors. Two weeks after injection, the mice were sacrificed and the DNA and RNA of the heart, liver, spleen, lung, and kidney was isolated. The expression of GFP and mCherry was determined in mRNA level by RT‐qPCR (Figure S6A–E). The expression frequency in mouse tissue was defined as the ratio of GFP to mCherry mRNA/cDNA copies in the tissue (Figure S6F). The copy numbers of GFP and mCherry vector genome in tissues were also detected (Figure S7A–E). The distribution frequency in each tissue was defined the same as expression frequency (Figure S7F). As we mixed every variant with AAV8 vector, we tested the ratio of vector genome copies (GFP/mCherry) in viral mixture. And the data of expression frequency and distribution frequency from DNA and RNA/cDNA in tissues were normalized to the above viral ratio for each variant.^58^

Compared with AAV8, five variants performed moderately higher level of transgene expression, especially in liver while showed no significant changes in their biodistribution profiles, with the highest viral particle count in the liver, similar to results from previous studies.^16^ Viral particles were also detected in other tissues such as the heart, spleen, lung, and kidney. Since AAVS3 is an AAV3B‐derived capsid with a poor transduction efficiency in mice, only the biodistribution of AAV8 mutants was detected.^59^

### 3.8 Evaluation of antibody profiles of the AAV8 and AAVS3 variants

**FIGURE 5:**
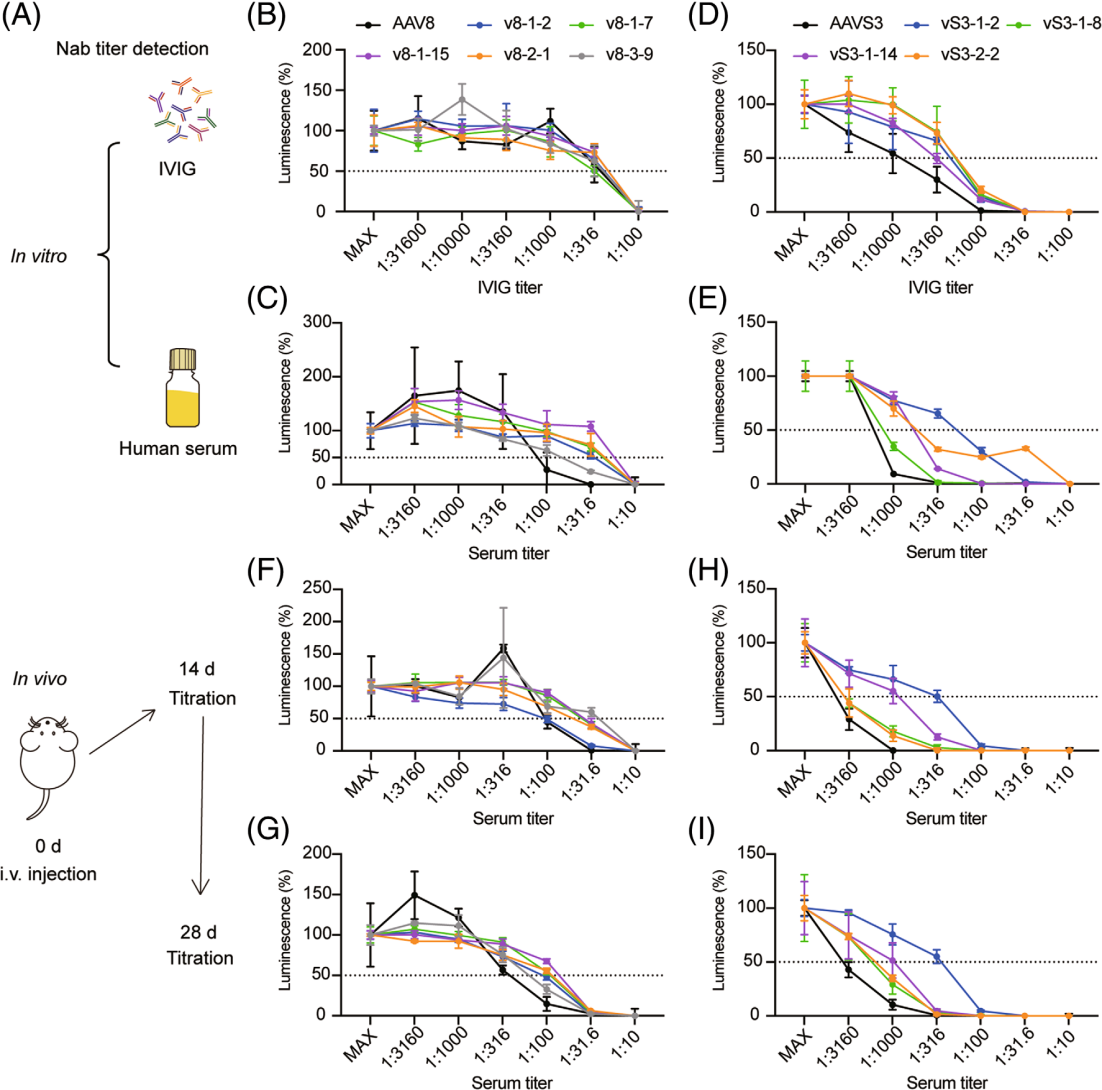
Validation of the neutralizing antibody profile in vitro and in vivo. (A) Workflow for analyzing the neutralizing antibody profile. (B‐C) AAV8 mutants and the parental AAV8 neutralizing antibody titer obtained using pooled IVIGs and human serum as the neutralization assay. (D‐E) AAVS3 mutants and the parental AAVS3 neutralizing antibody titer using pooled IVIGs and human serum as the neutralization assay. (F, G) The neutralizing activity against AAV8 vectors in AAV8‐treated mice 2 weeks (F) and 4 weeks (G) after treatment. (H‐I) The neutralizing activity against AAVS3 vectors in AAVS3‐treated mice 2 weeks (H) and 4 weeks (I) after treatment. Each data point represents the mean value of 3 replicates with SD

To evaluate whether our mutants had changed neutralizing antibody profiles and displayed a lower sensitivity towards pre‐existing neutralizing antibody, we analyzed these capacities under different conditions (Figure 5A).

We first performed in vitro experiments using pooled IVIGs and human serum. All five AAV8 mutants selected showed similar sensitivity with the parental AAV8 towards IVIGs and showed lower sensitivity in human serum. Particularly, the variant v8‐1‐15 showed the lowest sensitivity to the antibody (Figure 5B, C and Table S5). For AAVS3 mutants, all four mutants performed lower sensitivity than AAVS3 both for anti‐IVIGs and anti‐human serum, while the variant vS3‐1‐2 displayed the best properties in both conditions (Figure 5D, E and Table S5). Besides, we found that both for anti‐IVIGs and anti‐human serum, AAVS3 showed a higher sensitivity than AAV8, which was consistent with previous studies concluding that another AAV3B‐derived capsid, AAV‐LK03, had a higher sensitivity than AAV8.^60^

Additionally, we evaluated the in vivo antibody profile of our mutants compared with their parental AAV capsids. Three eight‐week‐old C57BL/6J male mice were injected intravenously with AAV8 and AAVS3. 2 and 4 weeks after injection, we separated the serum and detected the antibody titre. The results showed that compared with parental AAV8, all the mutants showed the ability to bypass the AAV8‐induced antibody profile, except for v8‐1‐2 at 2 weeks (Figure 5F, G and Table S6). For AAVS3 mutants, all four mutants showed the ability to bypass the AAVS3‐induced antibody profile at 2 and 4 weeks after injection, among which vS3‐1‐2 and vS3‐1‐14 showed the greatest difference with AAVS3 (Figure 5H, I and Table S6). Transgene expression was evaluated in AAV8‐treated mice to verify the changes in antibody profiles. We found higher level expression of transgene in AA8 pre‐treated mice reinjected with the mutants. These results confirm the altered antibody profile at the in vivo level of the mutant virus, which could escape the neutralizing antibodies produced by the parental virus (Figure S8).

In summary, all 5 AAV8 mutants performed better than AAV8 in human serum but showed similar sensitivity when pooled IVIGs were used for neutralization. Four of the 5 variants showed a different in vivo antibody profile compared with the parental AAV8 and all variants performed higher expression of transgene than parental AAV8 in AAV8‐pretreated mice. Among the AAVS3 mutants, all 4 variants had a better neutralizing antibody escaping capacity in IVIGs and human serum. For the in vivo antibody profile, all 4 mutants had different profiles compared with AAVS3, among which variants vS3‐1‐2 and vS‐1‐14 showed the greatest difference.

## 4 DISCUSSION

To date, 13 AAV serotypes and more than 150 AAV variants have been identified. However, as one of the delivery vectors for in vivo gene therapy, AAV still has some limitations. One of the most important problems is the immune response. Early clinical trials have shown that high‐dose AAV injections may lead to increased liver transaminases, indicating of liver inflammation and hepatoxicity.^26^ In addition, the prevalence of pre‐existing antibodies to AAV is high, although there are some regional variations.^61^, ^62^ Most of AAV engineering strategies only focus on improving the delivery efficiency of AAV^63^ or evading immune response.^64^ Therefore, we proposed a novel combined modification strategy to enhance both the properties of AAV. Glycosylation plays a key role in the viral life cycle, including binding to cellular receptors, internalizing, cytoplasmic transporting, and entering into the nucleus (reviewed in Ref. 65, 66). It has been shown that changing the glycosylation of the AAV2 capsid protein could increase the efficiency of its hepatic and ocular delivery, and the ability to escape the neutralizing antibodies was slightly enhanced.^37^ Endosomal escape might be one of the rate‐limiting steps in the AAV life cycle that limits the expression of the foreign genes.^67^, ^68^ rAAV vectors have to successfully escape endosomes and enter the nucleus or be degraded by the proteasome.^69^, ^70^ The PLA2‐like motif located in VP1u is thought to play a key role in endosomal escape of AAV and the activity is relatively low compared with other parvoviruses.^39^, ^40^, ^41^, ^42^ Whether the lower activity is related to the endosomal escape efficiency of AAV has been less studied. Based on the above principles, we hypothesized whether the combination of modifying the glycosylation of AAV capsids and enhancing the PLA2 activity could simultaneously improve the transduction efficiency and reduce immunogenicity.

In order to verify our proposal, we modified two clinically used liver‐targeting AAV vectors, AAV8 and AAVS3.^43^, ^44^, ^45^ Efficiency validation experiments in a variety of human‐derived tissue cell lines showed that the variants improved their gene expression in vitro. Although we used a liver cell line for screening, the variants showed increased efficiency in other human tissue cell lines. The PLA2 activity was detected, and it was modestly increased in variants with engineered PLA2 sites (v8‐2‐1, v8‐3‐9 and vS3‐2‐2) while most AAV virions could not be detected the PLA2 activity successfully. This may be due to the low PLA2 activity of AAV.^42^ We also detected whether N‐linked glycosylation had been changed on capsids of the variants by LC–MS/MS. We found the variants that designed to remove glycosylation (v8‐1‐15, v8‐3‐9, vS3‐1‐14 and vS3‐2‐2) met our expectations, while only vS3‐1‐2 (T451N) was added glycosylation at desired residue. Considering not all potential motifs would be glycosylated in vivo, the elimination of glycosylation might be easier to achieve.^55^, ^56^, ^57^ Besides, as posttranslational modifications (PTMs) can be various on AAV capsid proteins with different methods of packaging or packaging cell lines, further studies with different batch of AAV should be taken into consider.^71^, ^72^

Although five variants performed moderately higher level of transgene expression compared with AAV8, especially in liver, the biodistribution of the AAV8 mutants in C57BL/6J mice did not significantly change compared with the parental AAV8, indicating the mutated sites were not related to the tissue tropism. These results provided a broad insight for guiding the use of our mutants.

There were some possible limitations in our study. First, we did not directly cross‐compare the AAV8‐ and AAVS3‐derived engineered variants in vitro and in vivo due to the poor transduction in murine cells of AAV3B‐derived capsids.^59^ Therefore, we only compared the engineered variants with their parental AAVs. Second, our engineered variants showed elevated transduction efficiency on human cell lines, however, the variants did not significantly improve performance in mice. The modest improvement in vivo may be due to the use of human cell lines in the screening process, causing the species bias. This phenomenon has also been seen in other studies using humanized mice for screening.^73^ To test our hypothesis, several murine tissue cell lines were tested. Consistent with the results in vivo, the variants did not show higher transduction efficiency in these cell lines. Additional studies in nonhuman primates may facilitate the translation from in vitro selection systems to in vivo experiments with our AAV variants.

The wide range of prevalence of pre‐existing neutralizing antibodies against AAV vectors results in a major challenge for AAV as an in vivo gene delivery tool.^22^, ^23^, ^24^ Compared with AAV8, AAV3B‐derived AAVS3 may have a higher prevalence.^46^ The results of both IVIG and human serum showed that AAVS3 was more sensitive than AAV8 to pre‐existing neutralizing antibodies, while the mutants showed enhanced pre‐existing neutralizing antibody escape ability than their parental AAV. To further verify the changed antibody profiles of our mutants, we examined the transgene expression in vitro and in vivo. We found that the mutants could escape the parental AAV‐induced neutralizing antibody. For the AAV8 mutants, we also found higher level expression of foreign gene in vivo. This indicated that the antibody profiles of the mutants had been changed. Since the high titre of neutralizing antibody can hamper expression of the transgene in patients, these engineered AAV vectors might be dosed to the patients with high pre‐existing neutralizing antibody titre to the parental AAVs.^26^

Since a previous study of engineered AAV2 with glycosylation described that the modification slightly reduced the yield,^37^ to ensure that our mutants did not have increased costs of production, we also tested the production efficiency of our mutants. The viral copy numbers and the full/empty ratios were similar to the parental AAVs, demonstrating that our mutants would not increase manufacturing costs.

Our work screened several AAV variants based on AAV8 and AAVS3, two liver‐tropic variants. We engineered the parental AAVs and selected the candidates in human liver cells, obtaining several variants with higher transduction efficiency. This combined engineering strategy is universal and can be used in other conditions to obtain higher transduction AAV vectors with different tropism. This strategy, which influenced both the infectivity and immunological properties of AAV vectors, could engineer different AAV vectors in a simple and fast manner. Additionally, these variants with a higher transduction efficiency and lower sensitivity to neutralizing antibodies provided new candidates for preclinical and clinical trials. Our work provided more options for gene therapy delivery vectors, increasing the size of the available toolbox.

## AUTHOR CONTRIBUTIONS

Qi Zhou and Wei Li conceived the idea; Jiabao Han designed and performed experiments; Liyu Zhu and Jingwen Zhang helped AAV production; Lu Guo, Kai Xu and Xuehan Sun helped interpreted the data; Cheng Huang assisted with animal studies; Jiabao Han wrote the manuscript; Ying Zhang helped revise the manuscript. Qi Zhou and Wei Li acquired funding support and guided the whole study. All authors read and approved the final manuscript.

## CONFLICT OF INTEREST

The authors declare no conflict of interest.

## ETHICS STATEMENT

All experiments involving animals were approved by the Institutional Animal Use Committee of the Institute of Zoology, Chinese Academy of Sciences in Beijing.

## Supporting information


**APPENDIX S1** Supporting Information

## Data Availability

The data that support the findings of this study are available from the corresponding author upon reasonable request.
