# Supplementary material for: Rational engineering of adeno‐associated virus capsid enhances human hepatocyte tropism and reduces immunogenicity
Source: Cell Prolif. 2022 Sep 22;55(12):e13339. doi: 10.1111/cpr.13339 (PMC9715358; doi:10.1111/cpr.13339)
Supplement: Supplementary file 1 — APPENDIX S1 Supporting Information [file CPR-55-e13339-s001.docx]

**Supplementary Methods**

*Plasmids constructions with site-directed mutagenesis.* The overlapped forward and reverse primers with the flipped mutated sequences were designed and amplified by high-fidelity DNA polymerase (Phanta Flash Master Mix, Vazyme, P520). The product was cloned into pDP2/8 which was digested with *Swa*I and *Cla*I and transformed into Stbl3™ Chemically Competent E. coli. All mutants were sequenced to confirm the mutations.

*AAV titration.* AAV samples were treated with DNase I (Vazyme, DD4104) and Proteinase K (Takara, 9034). AAV samples were first digested by adding 2 μL 50 U/mL DNase I to 5 μL samples in 41 μL ddH_2_O, and incubated in a 37℃ dry bath for 1 hour. The samples were then inactivated by adding 2 μL 0.5M EDTA (Thermo Fisher Scientific, AM9260G) to each tube and incubated in a 70℃ dry bath for 10 min. Digested samples were then mixed with 2 μL Proteinase K and incubated at 70℃ for 30 min. Proteinase K was inactivated by incubation at 95℃ for 10 min. Treated virus samples (10 μL) were obtained, diluted (by 10-fold, 100-fold, and 1000-fold), and added into the 20 μL reaction system as templates. Each dilution was studied in triplicates. The standard curves in the 20 μL system were linear plasmids (digested with *Xho*I) of 1 × 10^12^, 1 × 10^11^, 1 × 10^10^, 1 × 10^9^, and 1 × 10^8^ copies, respectively. The qPCR reaction system was established according to the manufacturer’s instructions (AceQ Universal U + Probe Master Mix V2, Vazyme, Q513). Primer and probe sequences for GFP were GFP-F: TCCGCCACAACATCGAGGAC, GFP-R: GTAGTGGTTGTCGGGCAGCA, and GFP-P: 6-FAM-CAGCGTGCAGCTCGCCGACC-BHQ. The amplification was performed using QuantStudio 6 Flex real-time PCR systems as follows. Step 1: 37℃ 2 min, 1 cycle; step 2: 95℃ 10 min, 1 cycle; step 3: 95℃ 10 s, 65℃ 45 s, 40 cycles. The copy number of viral genomes was obtained by substituting the results into the standard curve.

*Generation of laboratory-grade purified AAVs.* The AAV mutant cassettes were CMV-Luciferase-GFP, and those of parental AAV were CMV-Luciferase-mCherry. The AAV precipitation solution was 40% PEG-8000 and 2.5 M NaCl in water. AAV lysis buffer was 150 mM NaCl, 50 mM Tris-HCl pH 8.5, and 2 mM MgCl_2_ in water. HEK293T cells were seeded 24 hours before transfection in 150-mm dishes to attain 80–90% confluency. The double-plasmid transient transfection system (6 μg expression plasmid and 24 μg packaging plasmid) was used with Lipofectamine™ LTX Reagent with PLUS™ Reagent (Thermo Fisher Scientific, 15338100), according to the manufacturer’s instructions. The supernatant of cells was collected 48 hours after transfection and were readded 25 mL of fresh medium. The supernatant of cells was collected 72 hours after transfection and mixed with that obtained after 48 hours. Cells were digested and lysed at 72 hours. We added 1:4 of the AAV precipitation solution volume to the supernatant, inverted tubes to mix, and stored overnight at 4℃ to precipitate proteins. The precipitate was centrifuged at 3,000 rpm for 30 min at 4℃ and resuspended in 10 mL AAV lysis buffer combined with the crude lysate. The mixture was digested by DNase I at a 1:10,000 ratio. Iodixanol density centrifugation was used for rAAV from crude lysate purifying.

*Mutation site combination iteration.* Mutations with higher gene expression than parental AAV8 or AAVS3 were combined, including two-site and three-site mutations. After constructing the plasmid and packaging the vectors, Huh7 cells were used for evaluating the transduction efficiency using the same detection method. For mutants with a higher transduction efficiency than that of parental AAV, and the experimental conditions and methods were consistent with the screening.

*LC-MS/MS detection*. About 10 μg sample was added DL-dithiothreitol (DTT) solution to a final concentration of 10 mmol/L, and incubated at 56℃ for 1 hour. Iodoacetamide (IAA) solution was added to a final concentration of 50 mmol/L and protected from light for 40 min. Trypsin was added at a weight ratio of 1:100 and incubated at 37℃ overnight. After digestion, the peptide was evaporated in a vacuum centrifuge at 45℃ and dissolved in the ^18^O water solution. PNGF enzyme was added at a weight ratio of 1:20 and incubated at 37℃ overnight. The peptide was evaporated and dissolved in the sample solution (0.1% formic acid, 2% acetonitrile), and the supernatant was transferred to the sample tube for mass spectrometry analysis. The parameters for LC/MS were showed below: Nanoflow UPLC: Easy-nLC 1200 system (ThermoFisher Scientific, USA), Nanocolumn: 150 μm×15 cm in-house made column packed with Acclaim PepMap, RPLC C18 (1.9 μm, 100 Å, Dr. Maisch GmbH, Germany), loaded sample volume: 5 μL, Mobile phase: A: 0.1% formic acid in water; B: 80% acetonitrile/0.1% formic acid in water, total flow rate: 600 nL/min, LC linear gradient: from 4% to 10% B for 5 min, from 10% to 22% B for 80 min, from 22% to 40% B for 25 min, from 40% to 95% B for 5 min and from 95% to 95% B for 5 min. The parameters for MS were showed below: spray voltage: 2.2 kV, capillary temperature: 270℃, MS resolution: 60000 at 400 m/z, MS precursor m/z range: 350.0-1500.0, activation type: HCD, normalized coll. energy: 30.0, activation time: 120.000. Up to top 20 most intense peptide ions from the preview scan in the Orbitrap.

*Evaluation of AAV mutants for human primary hepatocyte transduction efficiency experiments.* Human primary hepatocytes were purchased from bioIVT and thawed and plated according to the manufacturer’s instructions. Briefly, white/clear 96-well plates were coated with type I collagen 24 hours before plating. Cryoplateable human hepatocytes were thawed by INVITROGRO CP (bioIVT, Z99029) and plated onto collagen-coated white/clear 96-well plates at 7 × 10^4^ cells per well. After 2–4 hours, the plate was washed with complete INVITROGRO CP Medium (INVITROGRO CP Medium with 1% sp) and cultured with complete INVITROGRO HI Medium (INVITROGRO HI Medium (Z99009) with 1% Antibiotic-Antimycotic). The medium was exchanged every 24 hours after plating. Mutants and parental AAVs containing a CMV-Luciferase-GFP cassette were transfected 72 hours after plating at two MOIs (1 × 10^4^ and 1 × 10^5^) in triplicate. Cells were lysed 72 hours after transfection and their luciferase activity was detected using the luciferase assay kit according to the manufacturer’s instructions (Bright-Lite Luciferase Assay System, Vazyme, DD1204).

**Table S1**. Mutated sites of AAV8 variants.

| Name | Mutations |
| --- | --- |
| v8-1-1 | N273T |
| v8-1-2 | T332N |
| v8-1-3 | Y380N |
| v8-1-4 | Q452N |
| v8-1-5 | T460N |
| v8-1-6 | T493N |
| v8-1-7 | Q589N |
| v8-1-8 | Q260N |
| v8-1-9 | S710N |
| v8-1-10 | N14Q |
| v8-1-11 | N263Q |
| v8-1-12 | N338Q |
| v8-1-13 | N385Q |
| v8-1-14 | N499Q |
| v8-1-15 | N665Q |
| v8-2-1 | A98R |
| v8-2-2 | A98Q |
| v8-2-3 | A98E |
| v8-2-4 | F56G |
| v8-2-5 | T332N/Q589N |
| v8-2-6 | T332N/N665Q |
| v8-2-7 | Q589N/N665Q |
| v8-2-8 | T332N/Q589N/N665Q |
| v8-2-9 | A98R/T332N/Q589N |
| v8-2-10 | A98R/T332N/N665Q |
| v8-2-11 | A98R/Q589N/N665Q |
| v8-2-12 | A98R/T332N/Q589N/N665Q |
| v8-2-13 | A98Q/T332N/Q589N |
| v8-2-14 | A98Q/T332N/N665Q |
| v8-2-15 | A98Q/Q589N/N665Q |
| v8-2-16 | A98Q/T332N/Q589N/N665Q |
| v8-2-17 | A98E/T332N/Q589N |
| v8-2-18 | A98E/T332N/N665Q |
| v8-2-19 | A98E/Q589N/N665Q |
| v8-2-20 | A98E/T332N/Q589N/N665Q |
| v8-2-21 | F56G/T332N/Q589N |
| v8-2-22 | F56G/T332N/N665Q |
| v8-2-23 | F56G/Q589N/N665Q |
| v8-2-24 | F56G/T332N/Q589N/N665Q |
| v8-3-1 | A98R/T332N |
| v8-3-2 | A98R/Q589N |
| v8-3-3 | A98R/N665Q |
| v8-3-4 | A98Q/T332N |
| v8-3-5 | A98Q/Q589N |
| v8-3-6 | A98Q/N665Q |
| v8-3-7 | A98E/T332N |
| v8-3-8 | A98E/Q589N |
| v8-3-9 | A98E/N665Q |

**Table S2**. Mutated sites of AAVS3 variants.

| vS3-1-1 | T329N |
| --- | --- |
| vS3-1-2 | T451N |
| vS3-1-3 | S453N |
| vS3-1-4 | S490N |
| vS3-1-5 | N496T |
| vS3-1-6 | N500T |
| vS3-1-7 | Q585T |
| vS3-1-8 | Q585N |
| vS3-1-9 | S587N |
| vS3-1-10 | N335Q |
| vS3-1-11 | N382Q |
| vS3-1-12 | N446Q |
| vS3-1-13 | N457Q |
| vS3-1-14 | N497Q |
| vS3-1-15 | N706Q |
| vS3-2-1 | N457Q/N497Q |
| vS3-2-2 | A98R/N457Q |
| vS3-2-3 | A98R/N497Q/S162L |
| vS3-2-4 | A98R |
| vS3-2-5 | A98R/N457Q/N497Q |
| vS3-2-6 | A98Q/N457Q |
| vS3-2-7 | A98Q/N497Q/S162L |
| vS3-2-8 | A98Q |
| vS3-2-9 | A98Q/N457Q/N497Q |
| vS3-2-10 | A98E/N457Q |
| vS3-2-11 | A98E/N497Q/S162L |
| vS3-2-12 | A98E |
| vS3-2-13 | A98E/N457Q/N497Q |
| vS3-2-14 | A98R/N497Q |
| vS3-2-15 | A98Q/N497Q |
| vS3-2-16 | A98E/N497Q |

**Table S3**. Calculation of the sPLA2 activity for AAV8 and AAV8 variants.

| sPLA2 Activity (μmol/min/mL) | |
| --- | --- |
| AAV8 | ND |
| v8-1-2 | ND |
| v8-1-7 | ND |
| v8-1-15 | ND |
| v8-2-1 | 2.11E-03 |
| v8-3-9 | 4.22E-03 |

*ND means non-detected

**Table S4**. Calculation of the sPLA2 activity for AAVS3 and AAVS3 variants.

| sPLA2 Activity (μmol/min/mL) | |
| --- | --- |
| AAVS3 | ND |
| vS3-1-2 | ND |
| vS3-1-8 | ND |
| vS3-1-14 | ND |
| vS3-2-2 | 4.22E-03 |

*ND means non-detected

**Table S5**. Calculation of the *in vitro* Nab titers for AAV8 and AAVS3 variants.

| Sample | Nab titer | | Sample | Nab titer | |
| --- | --- | --- | --- | --- | --- |
|  | IVIG | Human serum |  | IVIG | Human serum |
| AAV8 | 1/344 | 1/135 | AAVS3 | 1/9563 | 1/1965 |
| v8-1-2 | 1/258 | 1/43 | vS3-1-2 | 1/1931 | 1/223 |
| v8-1-7 | 1/449 | 1/28 | vS3-1-8 | 1/1851 | 1/1087 |
| v8-1-15 | 1/258 | 1/13 | vS3-1-14 | 1/2856 | 1/456 |
| v8-2-1 | 1/392 | 1/29 | vS3-2-2 | 1/1740 | 1/432 |
| v8-3-9 | 1/332 | 1/81 |  |  |  |

**Table S6**. Calculation of the *in vivo* Nab titers for AAVS3 and AAVS3 variants.

| Sample | Nab titer | | Sample | Nab titer | |
| --- | --- | --- | --- | --- | --- |
|  | Two-week | Four-week |  | Two-week | Four-week |
| AAV8 | 1/90 | 1/311 | AAVS3 | >1/3160 | >1/3160 |
| v8-1-2 | 1/93 | 1/128 | vS3-1-2 | 1/302 | 1/308 |
| v8-1-7 | 1/33 | 1/115 | vS3-1-8 | >1/3160 | 1/2735 |
| v8-1-15 | 1/32 | 1/110 | vS3-1-14 | 1/963 | 1/1005 |
| v8-2-1 | 1/45 | 1/113 | vS3-2-2 | >1/3160 | 1/2614 |
| v8-3-9 | 1/28 | 1/137 |  |  |  |

**Figure S1**

**
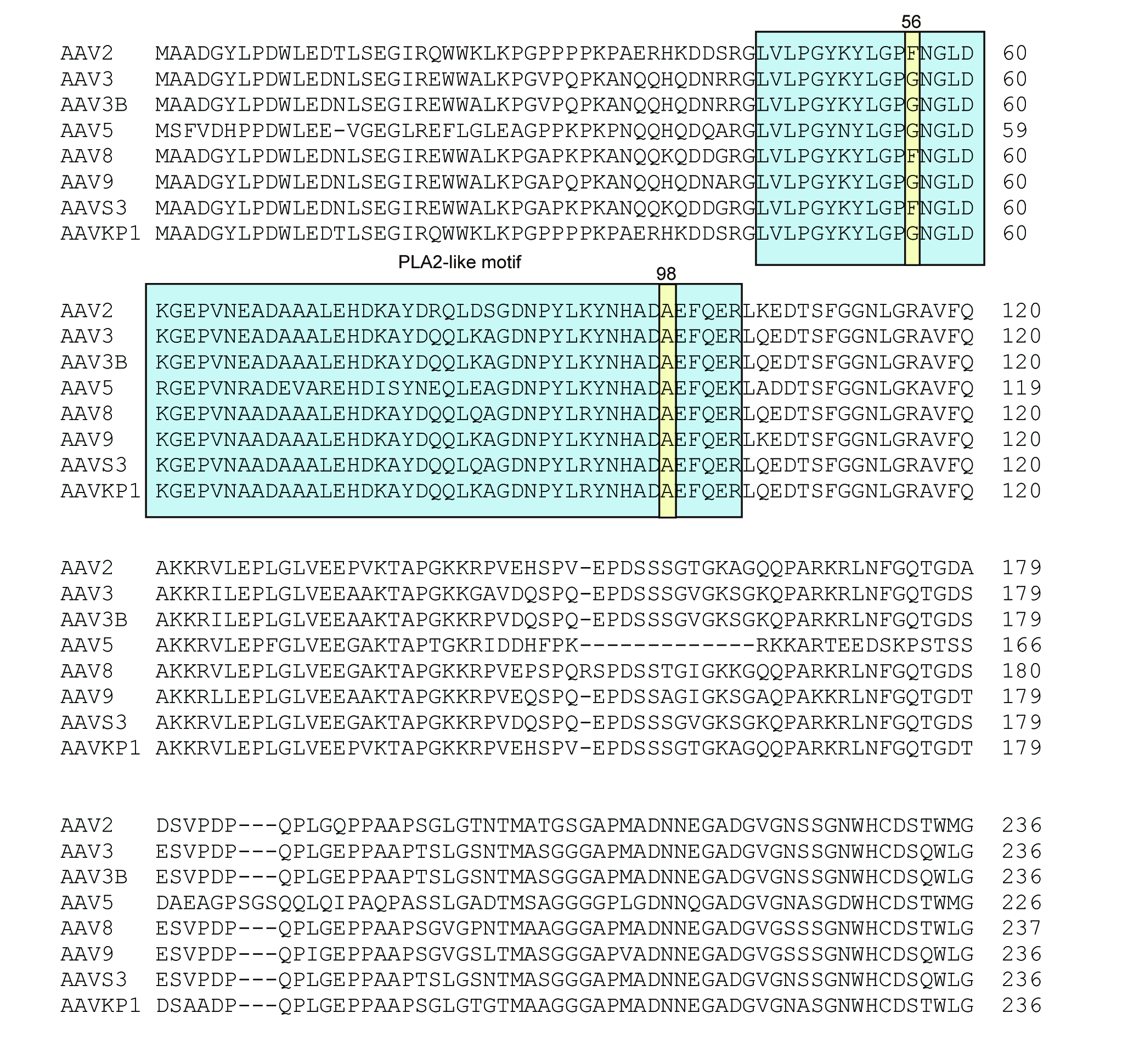
**

**Figure S1. Protein sequence alignment of VP1/2 N terminus.**

**Figure S2**


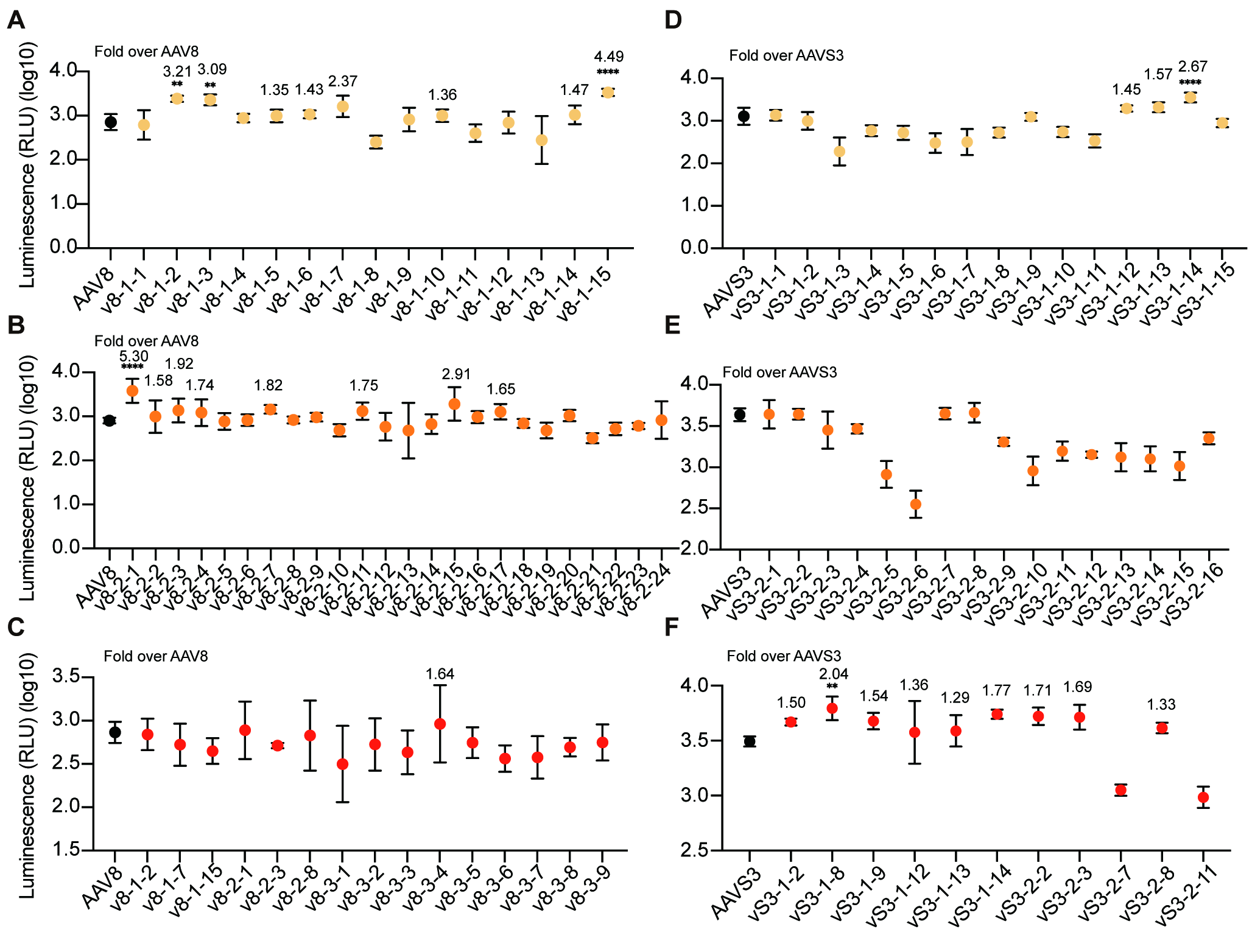


**Figure S2. Transduction of Huh7 cells.** (A-C) The three-round selections of AAV8 variants at an MOI of 10,000. (D-F) The three-round selections of AAVS3 variants at an MOI of 10,000. The Y-axis showed the intensity of the luminescence by relative luminescence units (RLU). Points represented the mean of 3 replicates and error bars represented the SD (standard deviation). Experimental values were analyzed via one-way ANOVA using Dunnett’s multiple comparison test and only statistically significant differences were indicated. ***P* < 0.01, *****P* < 0.0001.

**Figure S3**

**
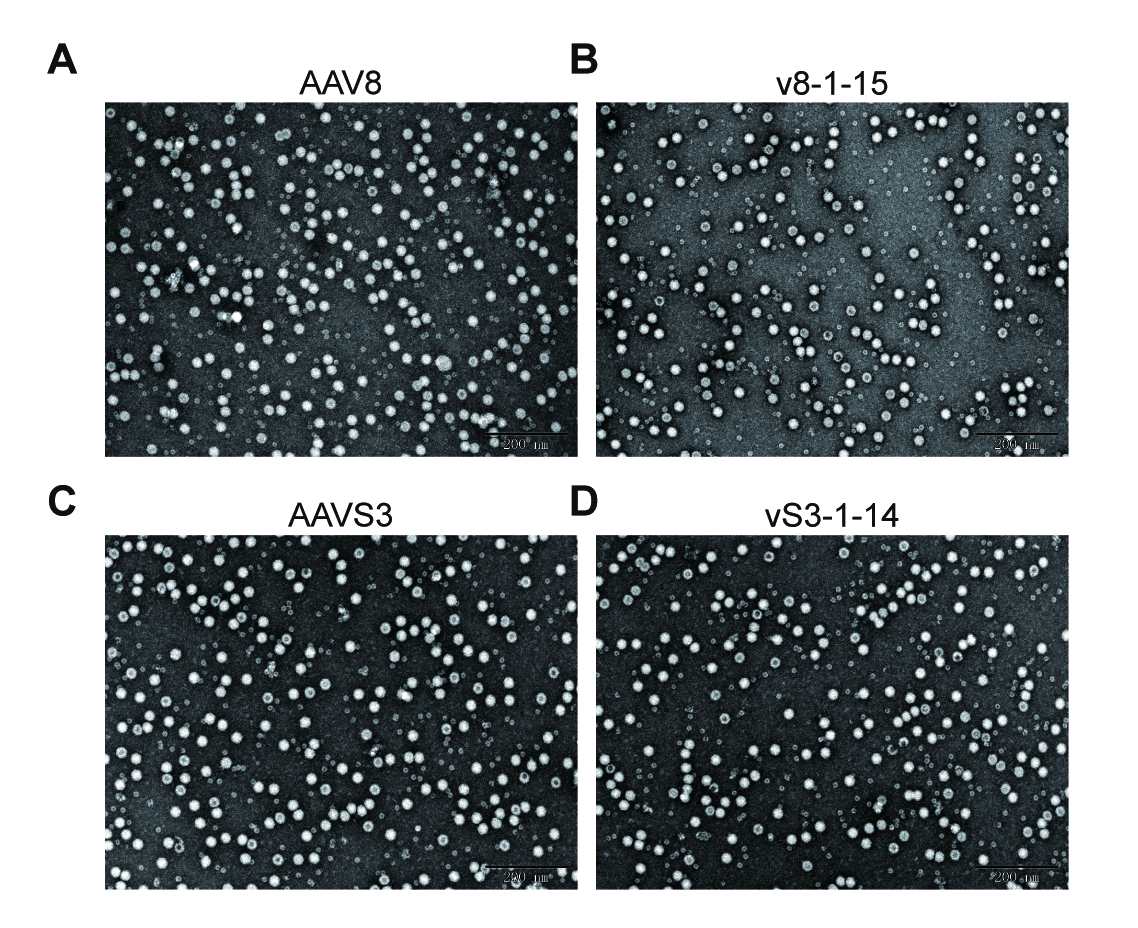
**

**Figure S3.** **Analysis of** **AAV vectors via negative-stain transmission electron microscopy.** (A) AAV8, (B) v8-1-15, (C) AAVS3 and (D) vS3-1-14.

**Figure S4**

**
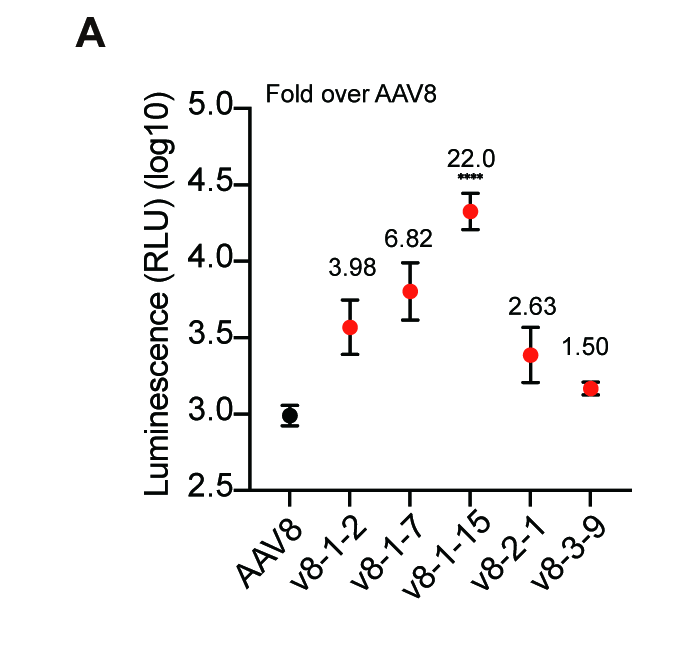
**

**Figure S4. Transduction of human primary hepatocytes.** Human primary hepatocytes were transduced with the different AAV8 variants at an MOI of 1,000,000. The Y-axis showed the intensity of the luminescence by relative luminescence units (RLU). Points represented the mean of 3 replicates and error bars represented the SD. Experimental values were analyzed via one-way ANOVA using Dunnett’s multiple comparison test and only statistically significant differences were indicated. *****P* < 0.0001.

**Figure S5**


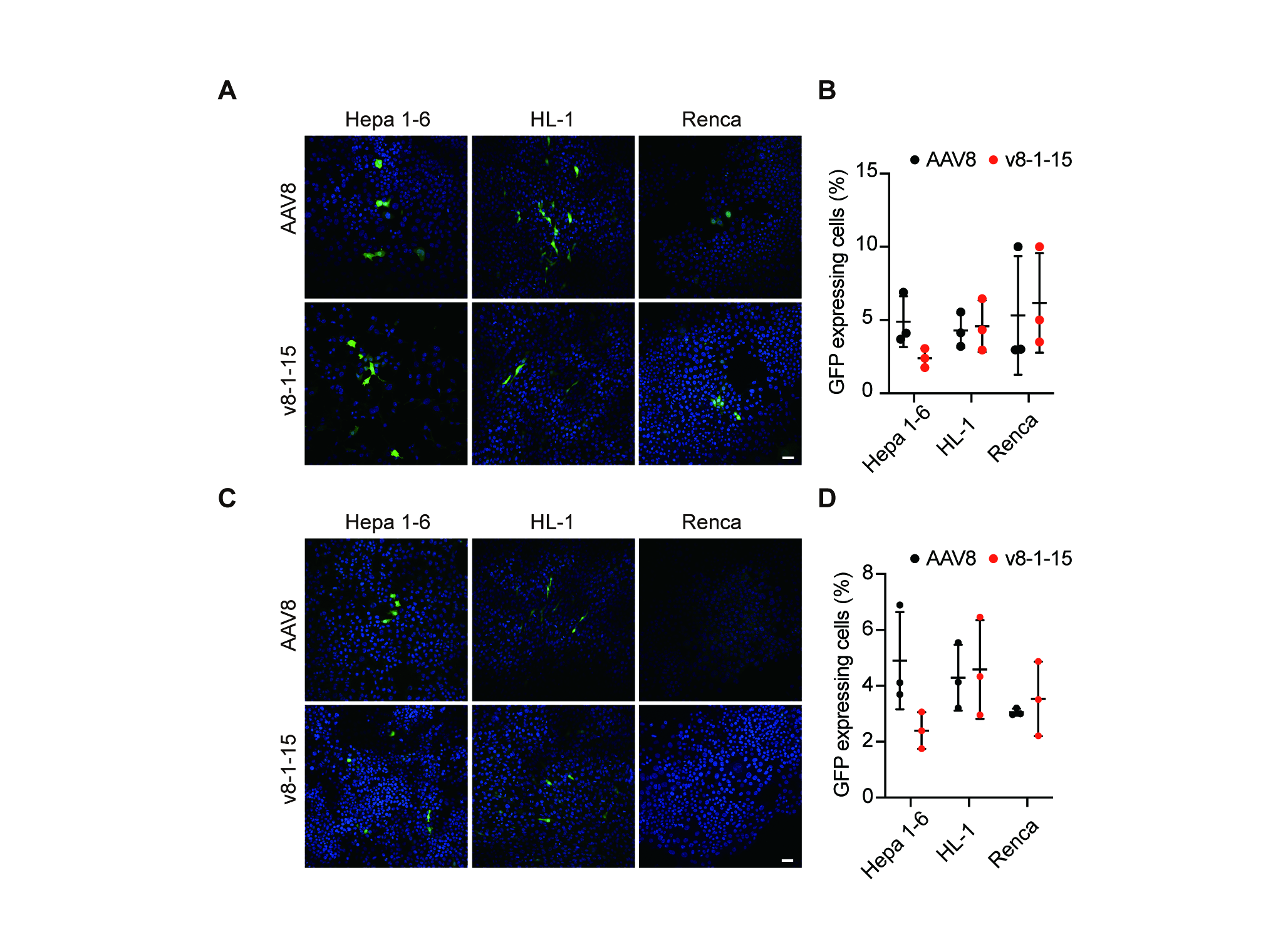


**Figure S5. Transduction of multiple mouse-derived tissue cell lines.** (A-B) Hepa 1-6 and HL-1 were transduced with different vectors at an MOI of 10,000 and the quantification of transduction efficiency was calculated. (C-D) Cells were also transduced at an MOI of 100,000 and quantified the efficiency. Each data point represented an area for each sample. Points represented the mean of 3 replicates and error bars represented SD. Experimental values were analyzed via 2-way ANOVA using Sidak’s multiple comparison test.

**Figure S6**

**
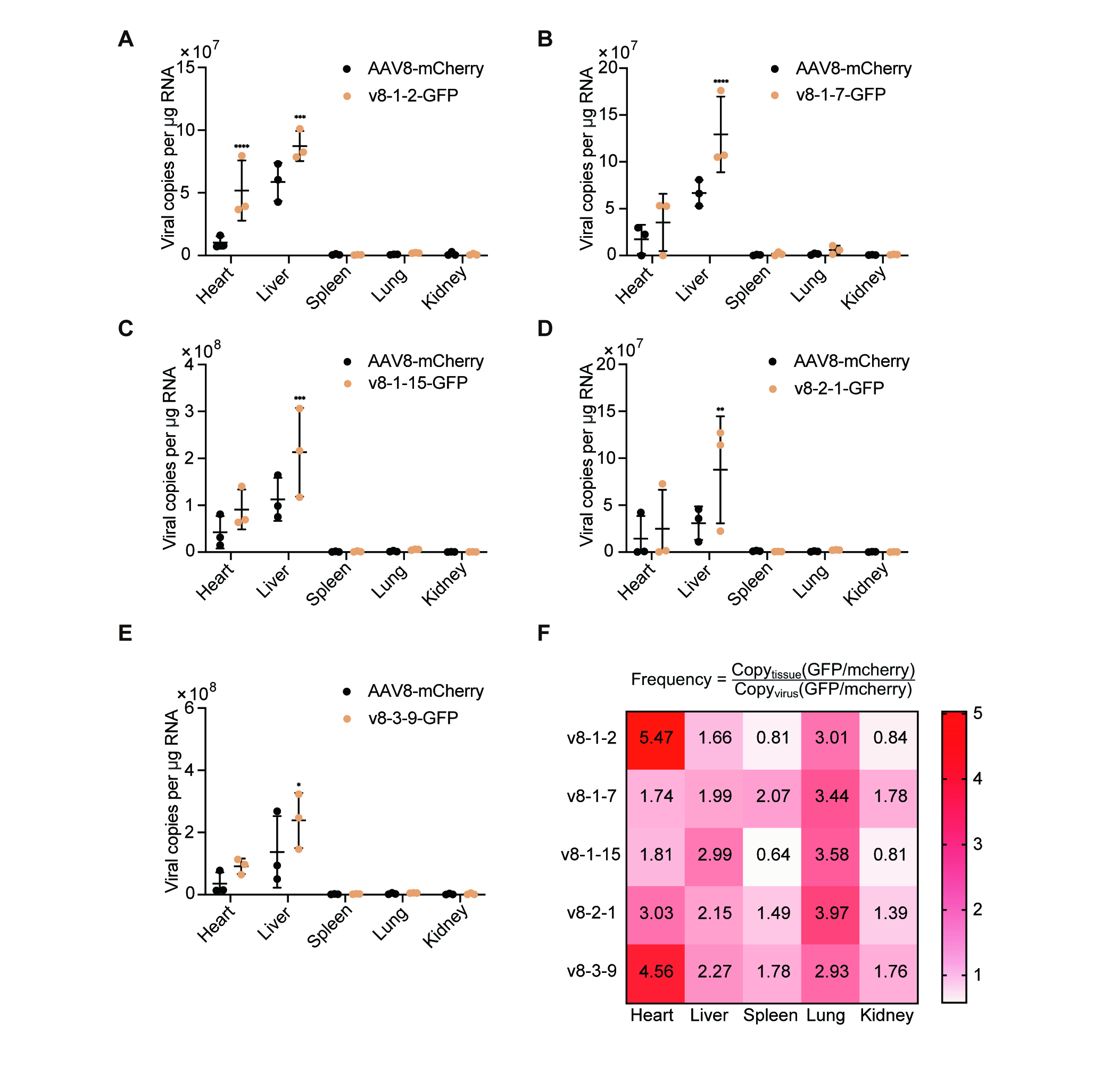
**

**Figure S6. Transgene expression of *in vivo* biodistribution of the AAV8 mutants in C57BL/6J mice.** (A-E) RNA copies of GFP and mCherry in different tissues were detected for the variants v8-1-2, v8-1-7, v8-1-15, v8-2-1 and v8-3-9. (F) Viral frequency was defined as the ratio of GFP/mCherry mRNA/cDNA copies in tissue to viral mixture genome. Each data point was represented a copy number of an animal and was showed the mean of 3 replicate mice. Experimental values were analyzed via two-way ANOVA using Sidak’s multiple comparison test and only statistically significant differences were indicated. **P* < 0.05, ***P* < 0.01, ****P* < 0.001, *****P* < 0.0001.

**Figure S7**


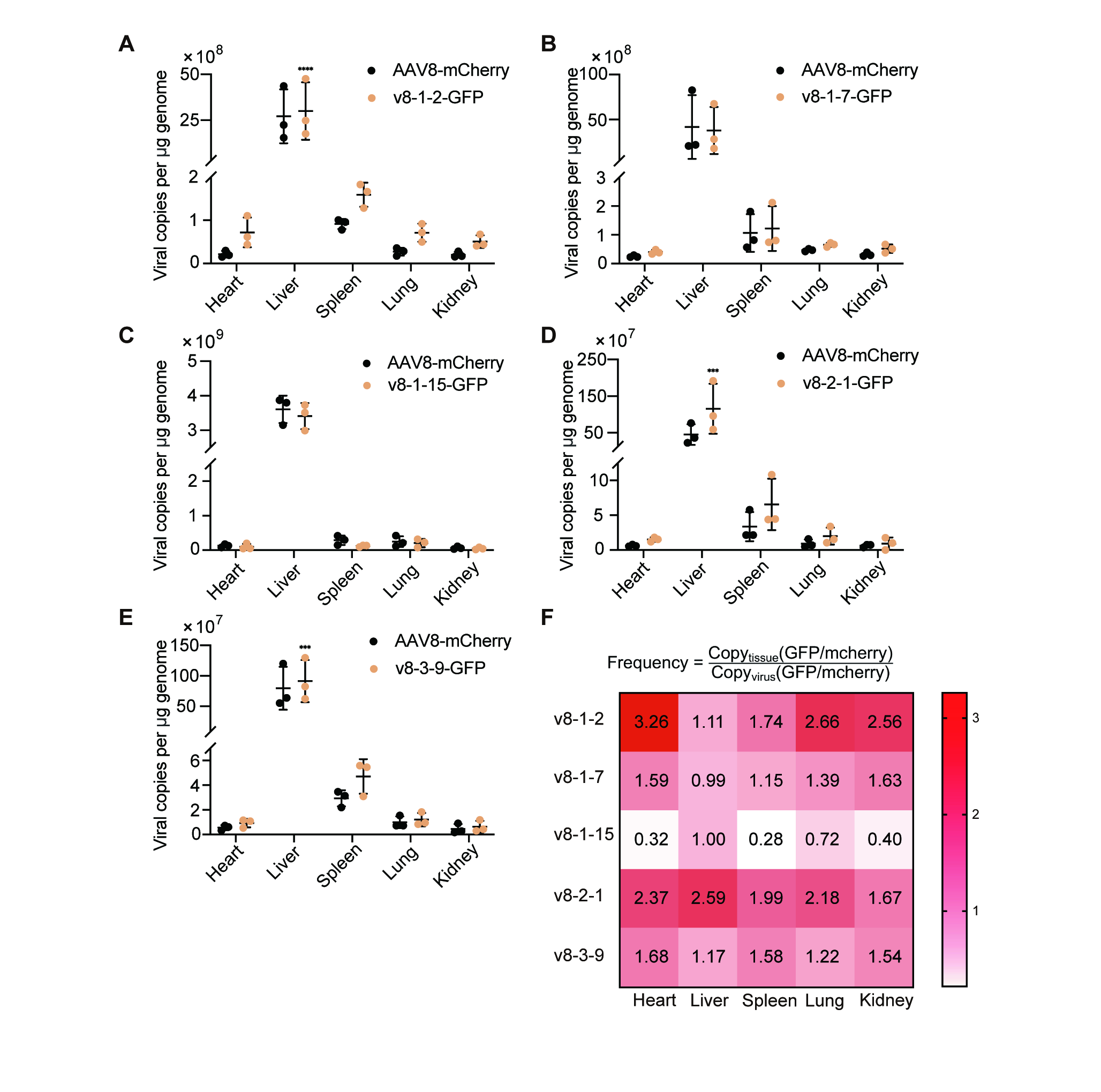


**Figure S7. Genoemic copies of *in vivo* biodistribution of the AAV8 mutants in C57BL/6J mice.** (A-E) Genomic copies of GFP and mCherry in different tissues were detected for the variants v8-1-2, v8-1-7, v8-1-15, v8-2-1 and v8-3-9. (F) Viral frequency was defined as the ratio of GFP/mCherry genome copies in tissue to viral mixture. Each data point was represented a copy number of an animal and was showed the mean of 3 replicate mice. Experimental values were analyzed via two-way ANOVA using Sidak’s multiple comparison test and only statistically significant differences were indicated. ****P* < 0.001, *****P* < 0.0001.

**Figure S8**

**
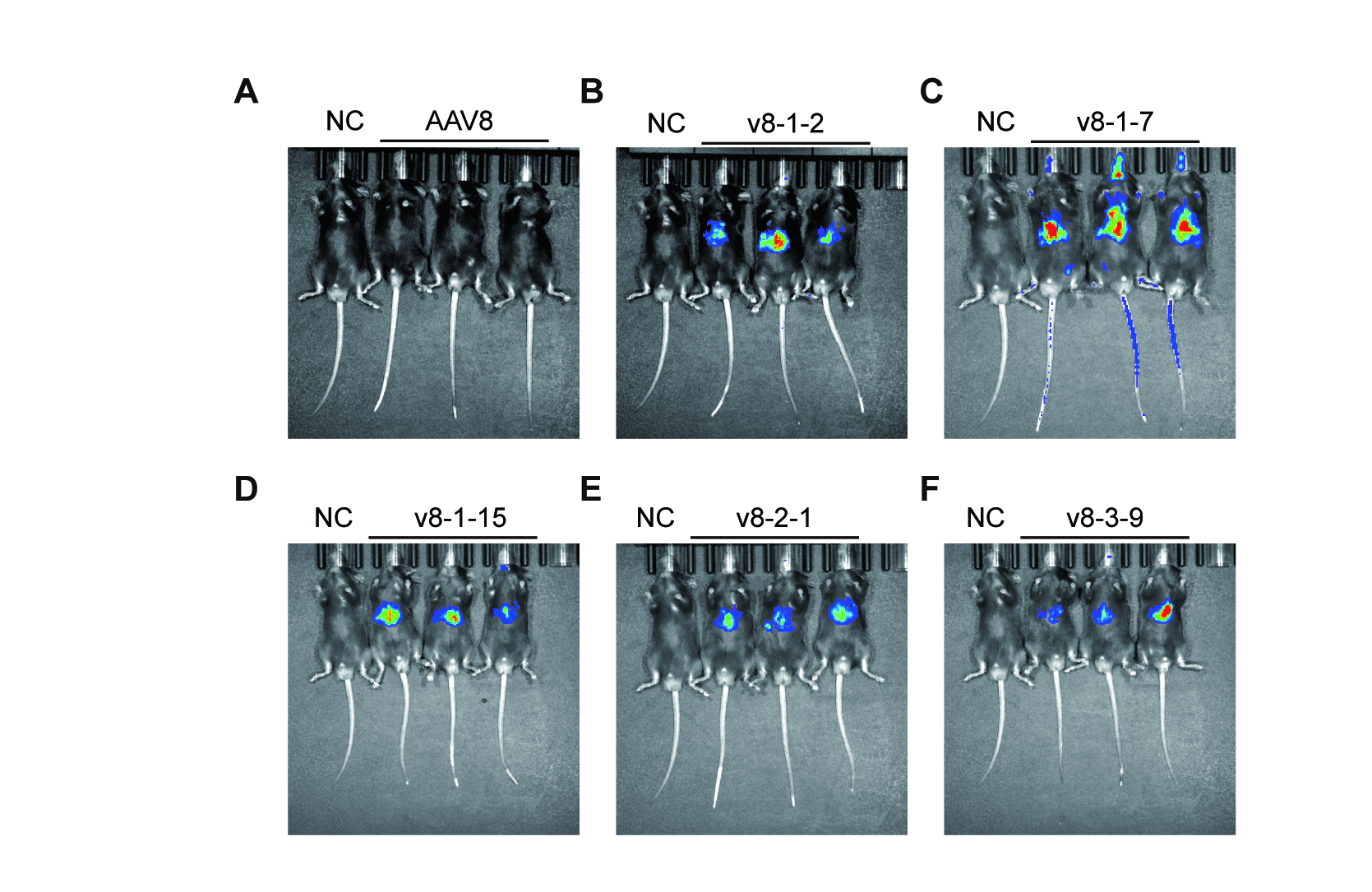
**

**Figure S8. *In vivo* bioluminescence imaging of firefly luciferase in AAV8 pre-treated mice.** Bioluminescence was monitored one week after reinjected (A) AAV8, (B) v8-1-2, (C) v8-1-7, (D) v8-1-15, (E) v8-2-1 and (F) v8-3-9.
